# Supplementary figures and images for: Single-view 3D reconstruction via dual attention
Source: PeerJ Comput Sci. 2024 Oct 22;10:e2403. doi: 10.7717/peerj-cs.2403 (PMC11623020; doi:10.7717/peerj-cs.2403)

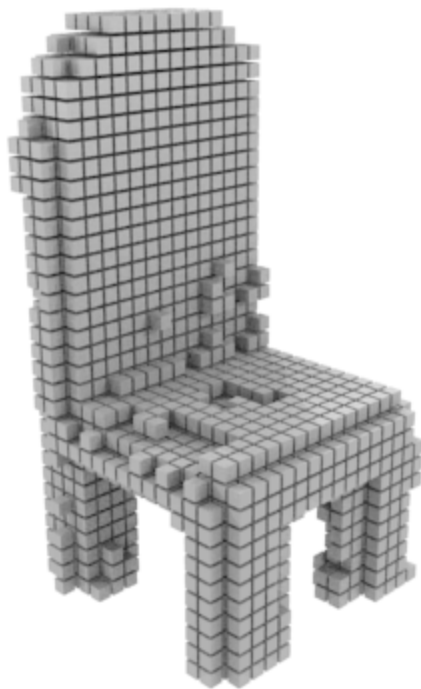

Supplement: Supplemental Information 1 [file peerj-cs-10-2403-s001.zip › result_from_3dretr/cs-100114-3dretr3b3a9f4e3aa9f2f4d39a194653571dfc.pdf]

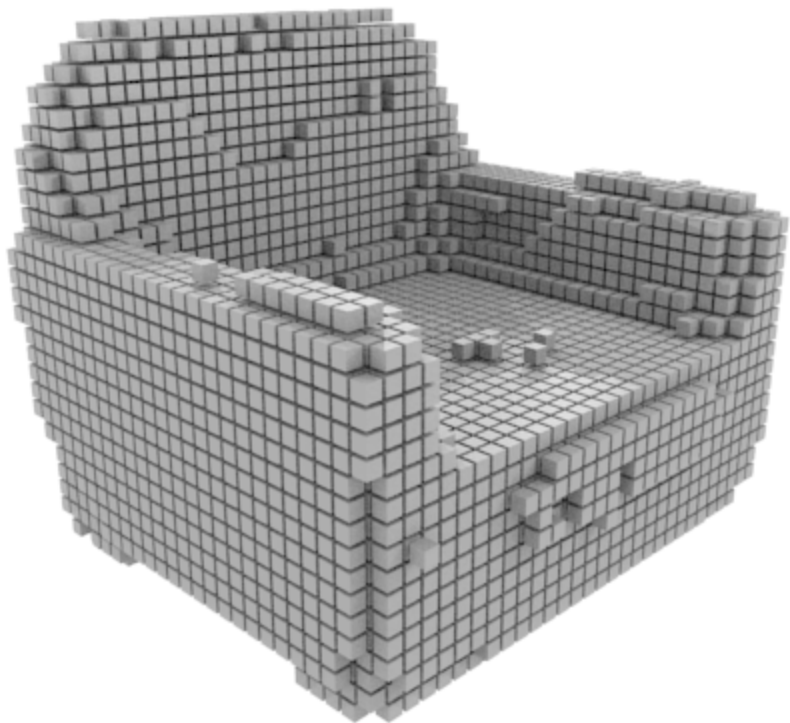

Supplement: Supplemental Information 1 [file peerj-cs-10-2403-s001.zip › result_from_3dretr/cs-100114-3dretrbe4c88a130e622a21961e650f3cfa396.pdf]

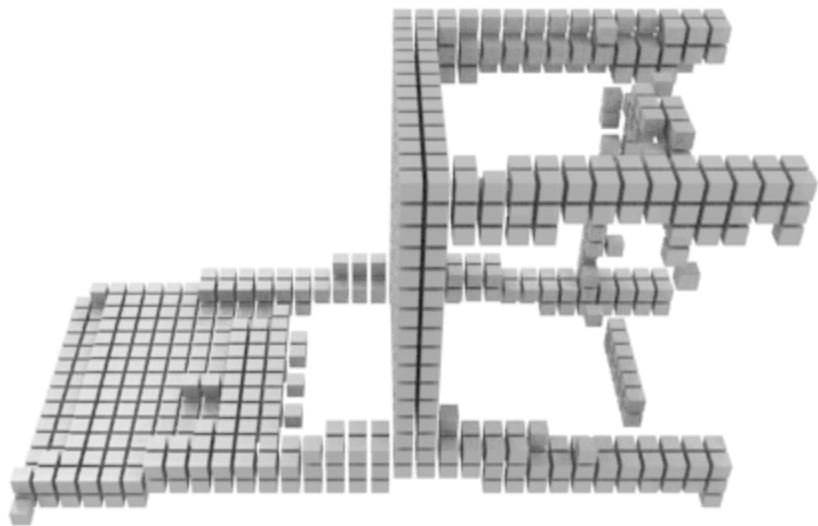

Supplement: Supplemental Information 1 [file peerj-cs-10-2403-s001.zip › result_from_3dretr/cs-100114-3dretrchair.pdf]

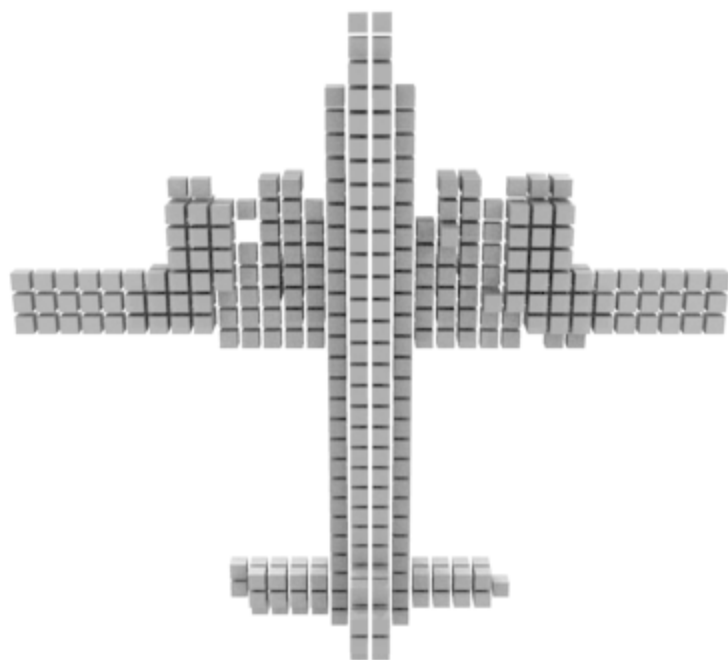

Supplement: Supplemental Information 1 [file peerj-cs-10-2403-s001.zip › result_from_3dretr/cs-100114-3dretrplane.pdf]

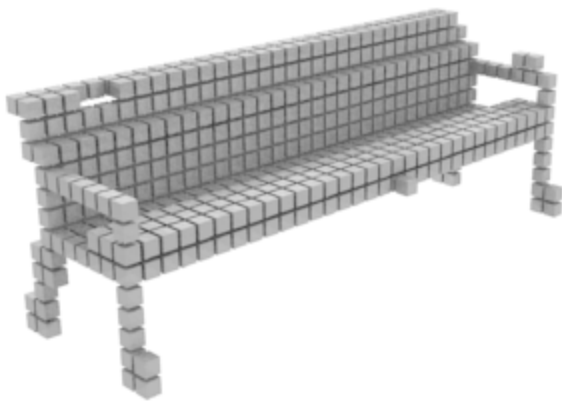

Supplement: Supplemental Information 1 [file peerj-cs-10-2403-s001.zip › result_from_3dretr/cs-100114-3dretr_longchair.pdf]

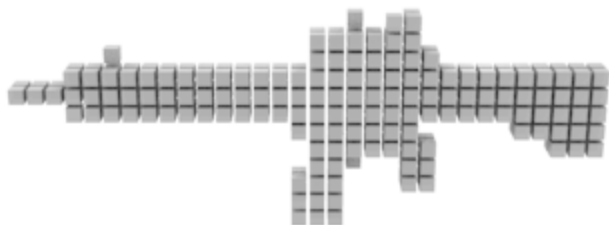

Supplement: Supplemental Information 1 [file peerj-cs-10-2403-s001.zip › result_from_3dretr/cs-100114-3dretr_rifle.pdf]

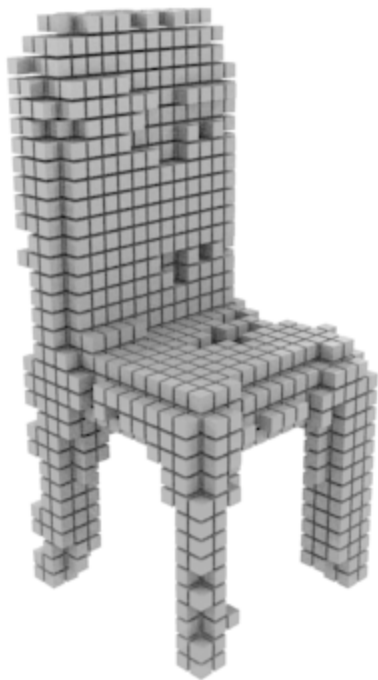

Supplement: Supplemental Information 2 [file peerj-cs-10-2403-s002.zip › result_from_pix2vox/cs-100114-pix2vox++3b3a9f4e3aa9f2f4d39a194653571dfc.pdf]

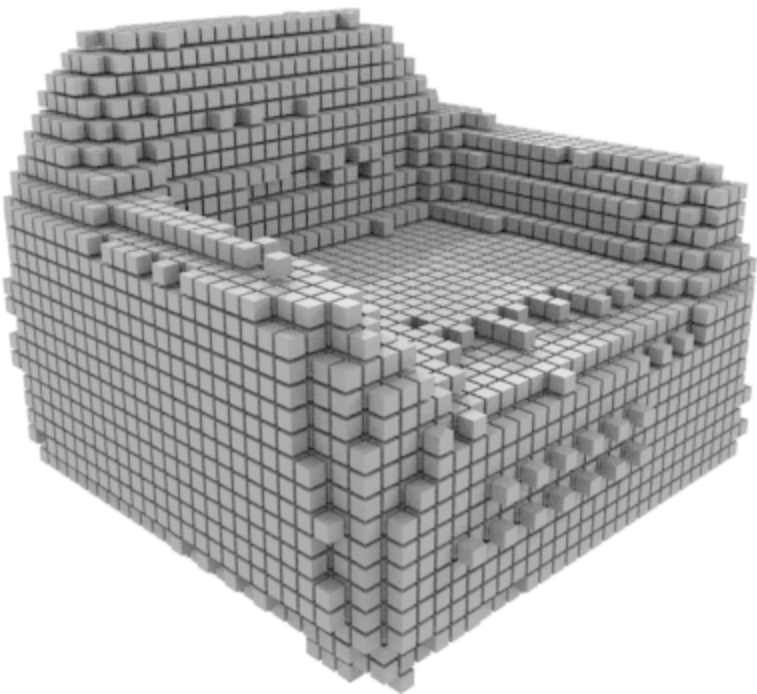

Supplement: Supplemental Information 2 [file peerj-cs-10-2403-s002.zip › result_from_pix2vox/cs-100114-pix2vox++be4c88a130e622a21961e650f3cfa396.pdf]

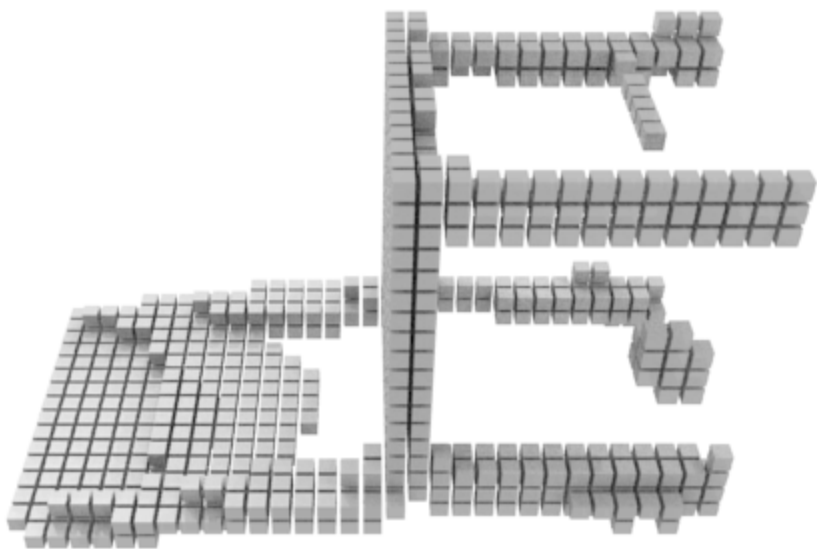

Supplement: Supplemental Information 2 [file peerj-cs-10-2403-s002.zip › result_from_pix2vox/cs-100114-pix2vox++chair.pdf]

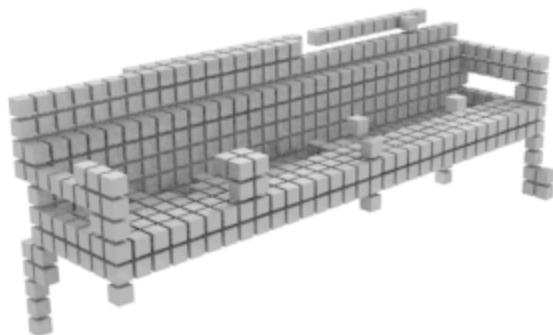

Supplement: Supplemental Information 2 [file peerj-cs-10-2403-s002.zip › result_from_pix2vox/cs-100114-pix2vox++longchair.pdf]

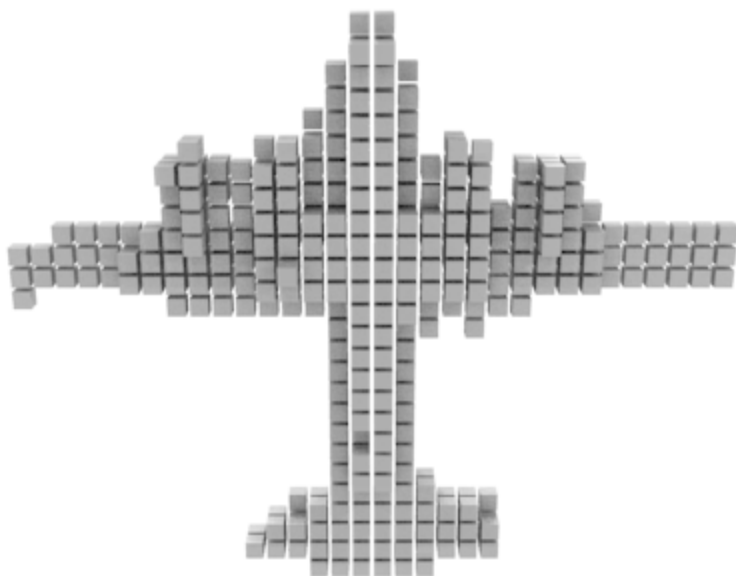

Supplement: Supplemental Information 2 [file peerj-cs-10-2403-s002.zip › result_from_pix2vox/cs-100114-pix2vox++plane.pdf]

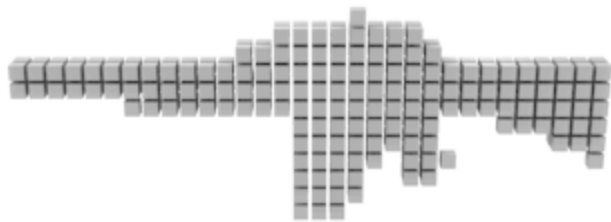

Supplement: Supplemental Information 2 [file peerj-cs-10-2403-s002.zip › result_from_pix2vox/cs-100114-pix2vox++_rifle.pdf]

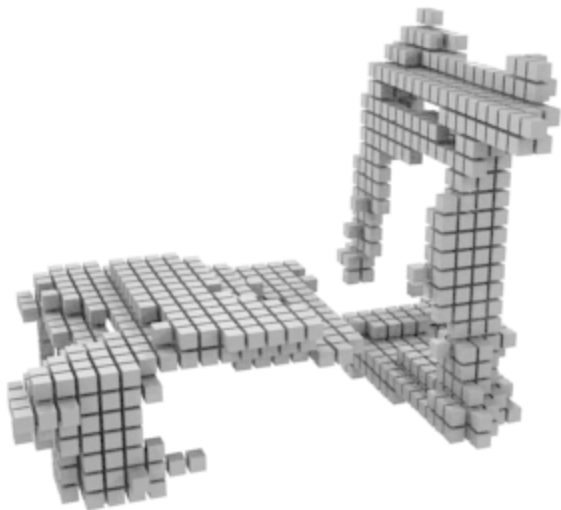

Supplement: Supplemental Information 2 [file peerj-cs-10-2403-s002.zip › result_from_pix2vox/cs-100114-pix3d_pix2vox++0.pdf]

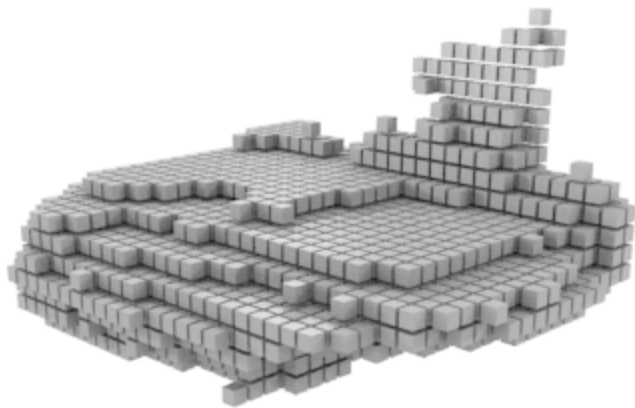

Supplement: Supplemental Information 2 [file peerj-cs-10-2403-s002.zip › result_from_pix2vox/cs-100114-pix3d_pix2vox++1.pdf]

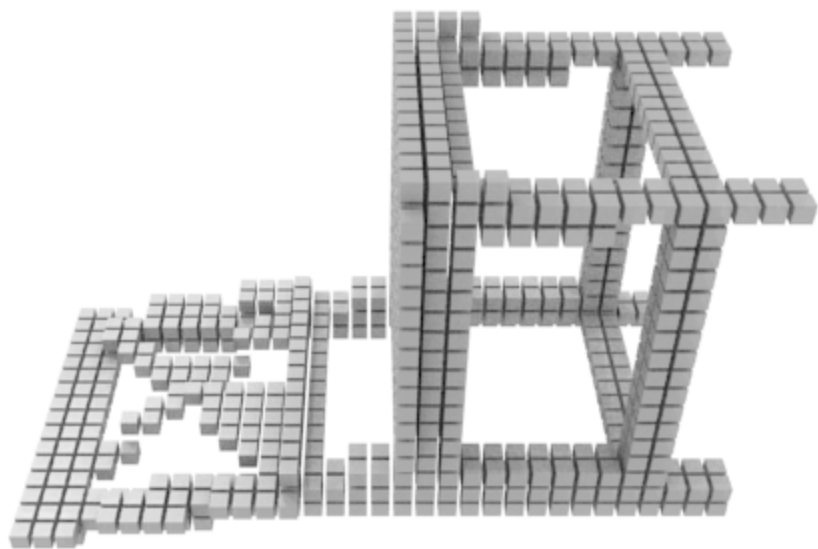

Supplement: Supplemental Information 3 [file peerj-cs-10-2403-s003.zip › GroundTruth/cs-100114-chair_gt.pdf]

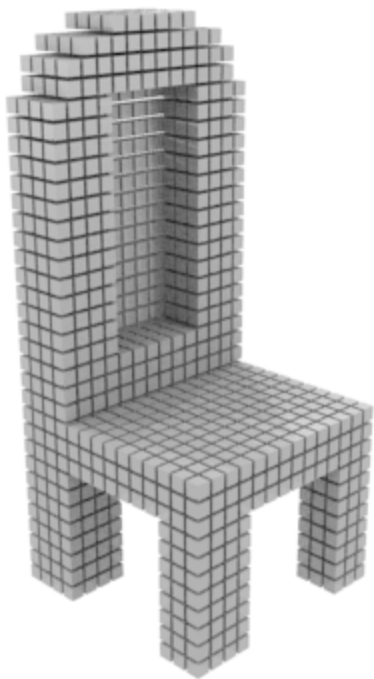

Supplement: Supplemental Information 3 [file peerj-cs-10-2403-s003.zip › GroundTruth/cs-100114-GT3b3a9f4e3aa9f2f4d39a194653571dfc.pdf]

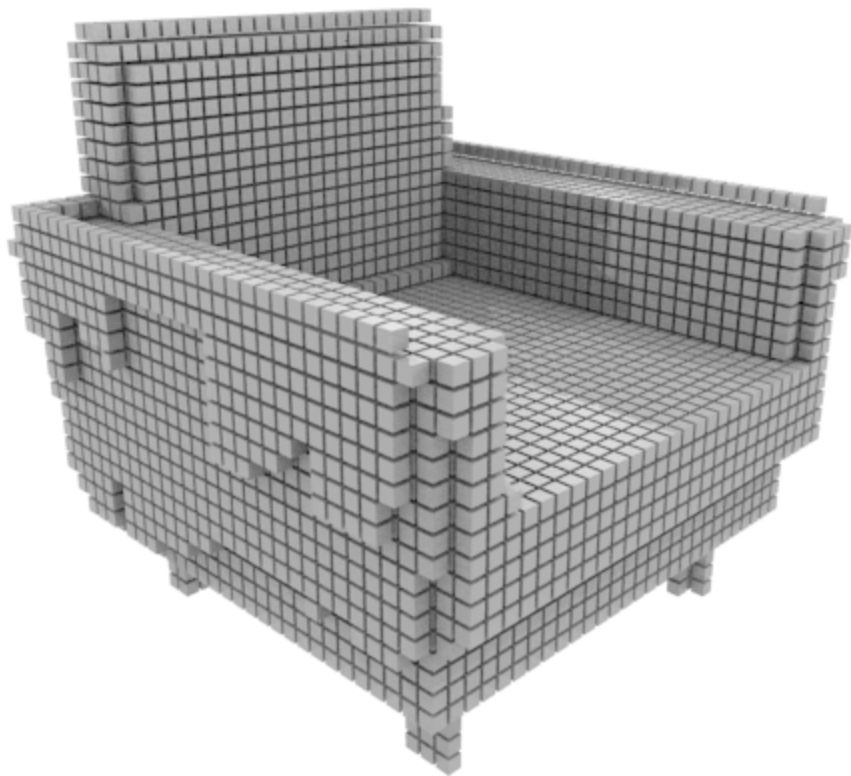

Supplement: Supplemental Information 3 [file peerj-cs-10-2403-s003.zip › GroundTruth/cs-100114-GTbe4c88a130e622a21961e650f3cfa396.pdf]

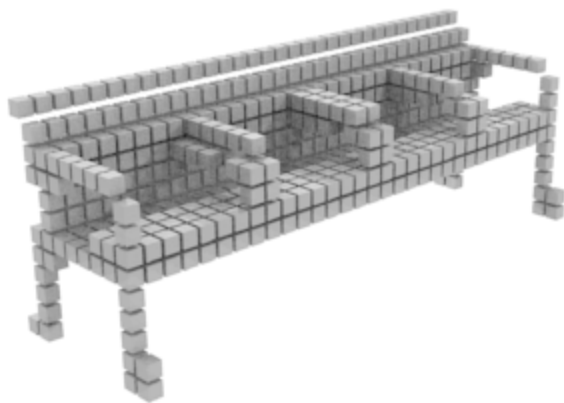

Supplement: Supplemental Information 3 [file peerj-cs-10-2403-s003.zip › GroundTruth/cs-100114-GT_longchair.pdf]

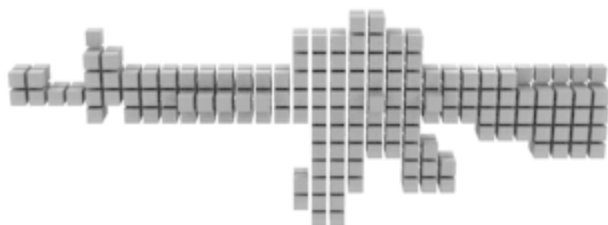

Supplement: Supplemental Information 3 [file peerj-cs-10-2403-s003.zip › GroundTruth/cs-100114-GT_rifle.pdf]

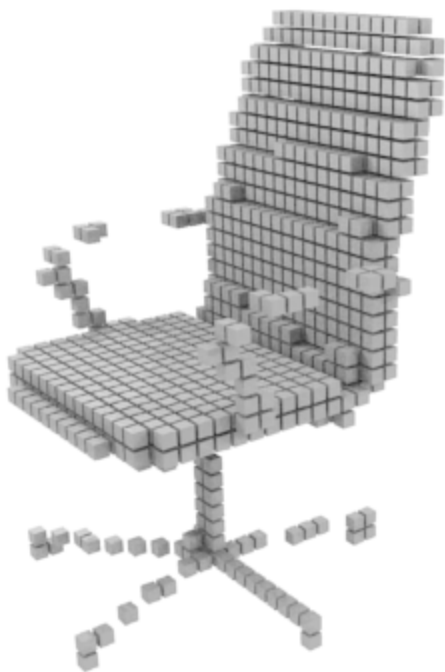

Supplement: Supplemental Information 3 [file peerj-cs-10-2403-s003.zip › GroundTruth/cs-100114-pix3d_GT00.pdf]

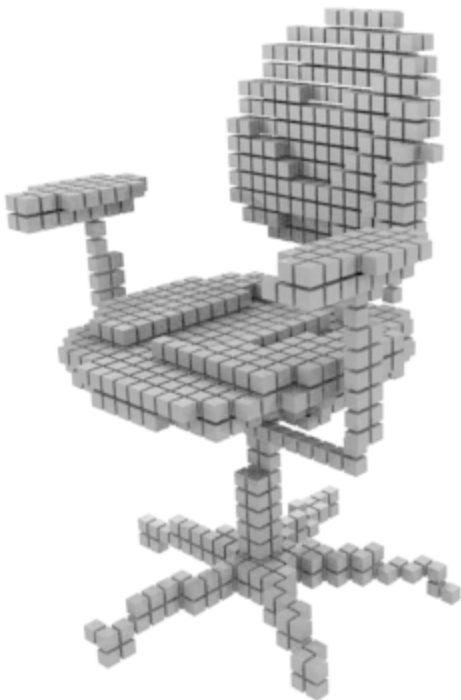

Supplement: Supplemental Information 3 [file peerj-cs-10-2403-s003.zip › GroundTruth/cs-100114-pix3d_GT01.pdf]

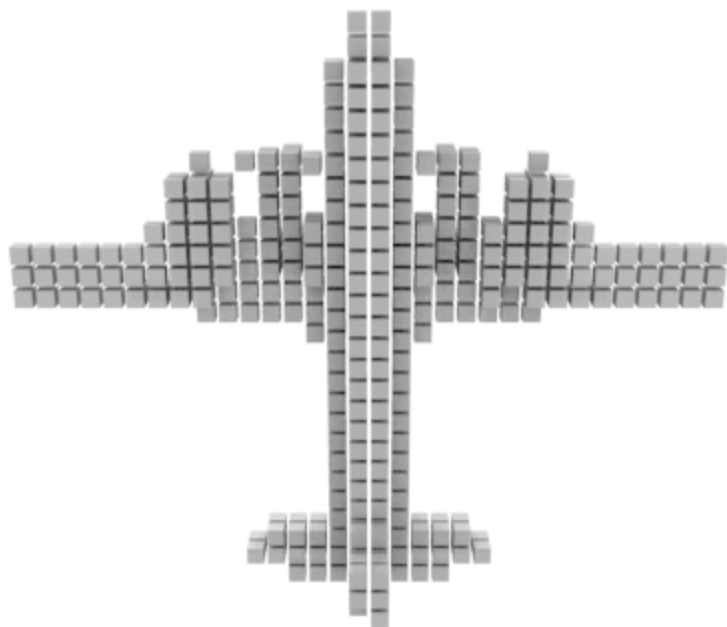

Supplement: Supplemental Information 3 [file peerj-cs-10-2403-s003.zip › GroundTruth/cs-100114-plane_gt.pdf]

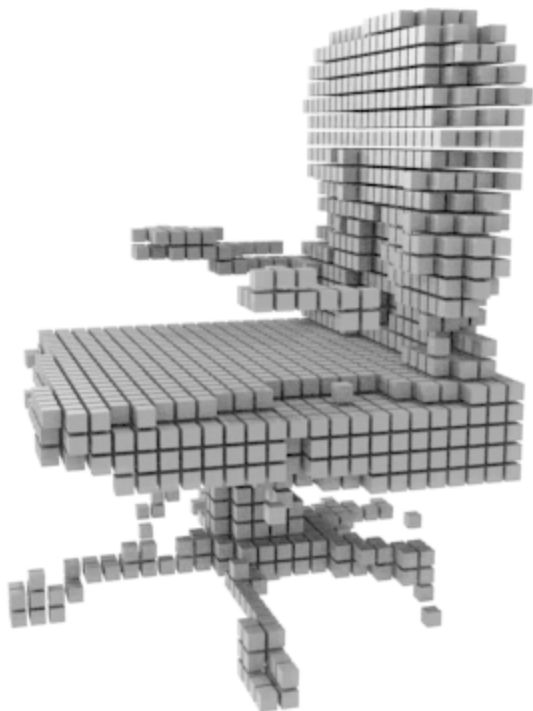

Supplement: Supplemental Information 4 [file peerj-cs-10-2403-s004.zip › ResultFromOurMethod/cs-100114-pix3d_R3DSWIN_00.pdf]

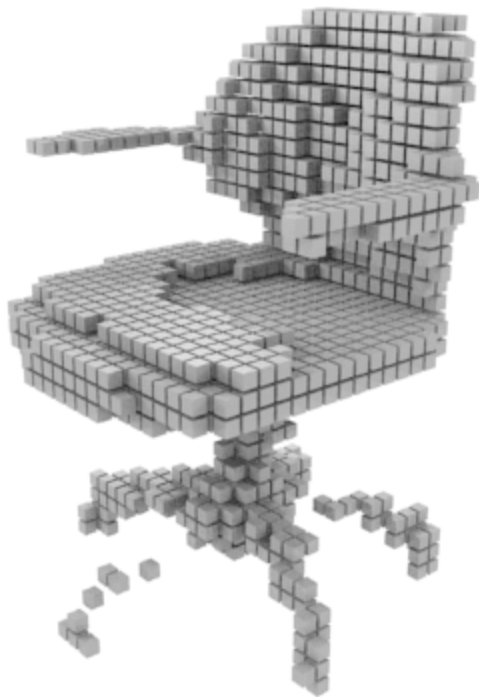

Supplement: Supplemental Information 4 [file peerj-cs-10-2403-s004.zip › ResultFromOurMethod/cs-100114-pix3d_R3DSWIN_01.pdf]

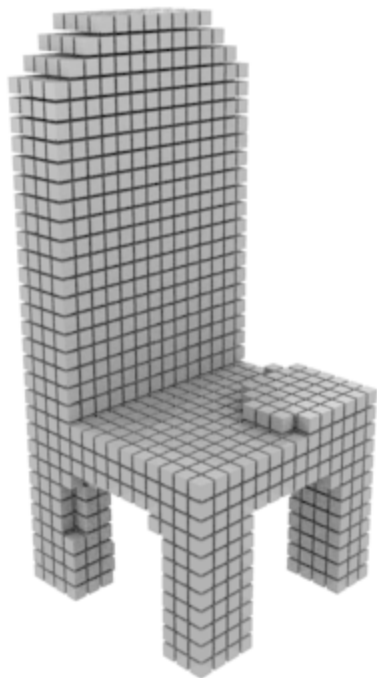

Supplement: Supplemental Information 4 [file peerj-cs-10-2403-s004.zip › ResultFromOurMethod/cs-100114-R3Davit3b3a9f4e3aa9f2f4d39a194653571dfc.pdf]

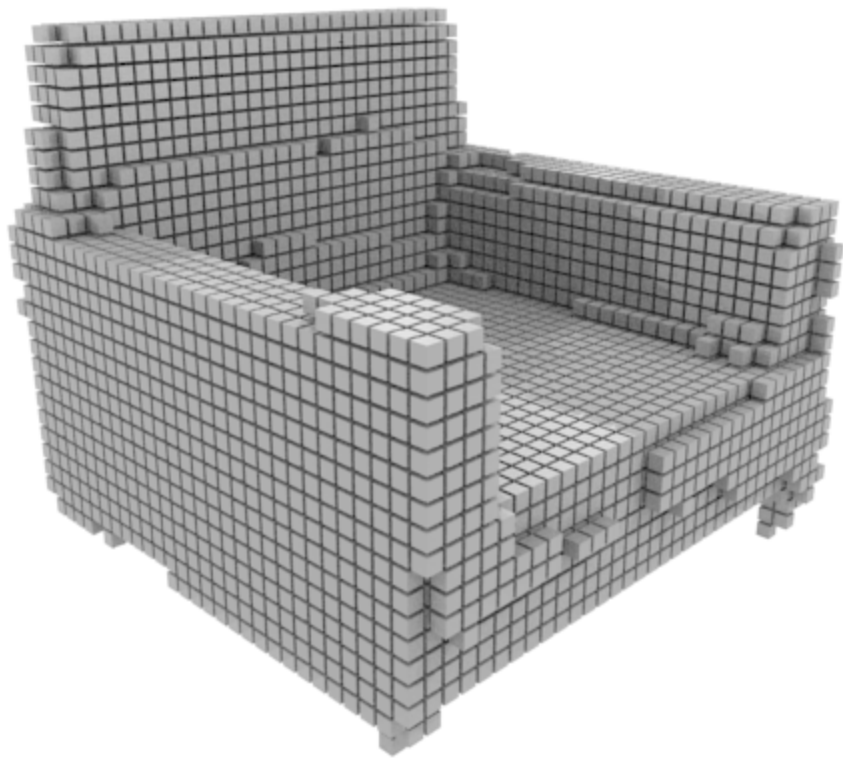

Supplement: Supplemental Information 4 [file peerj-cs-10-2403-s004.zip › ResultFromOurMethod/cs-100114-R3Davitbe4c88a130e622a21961e650f3cfa396.binvox.pdf]

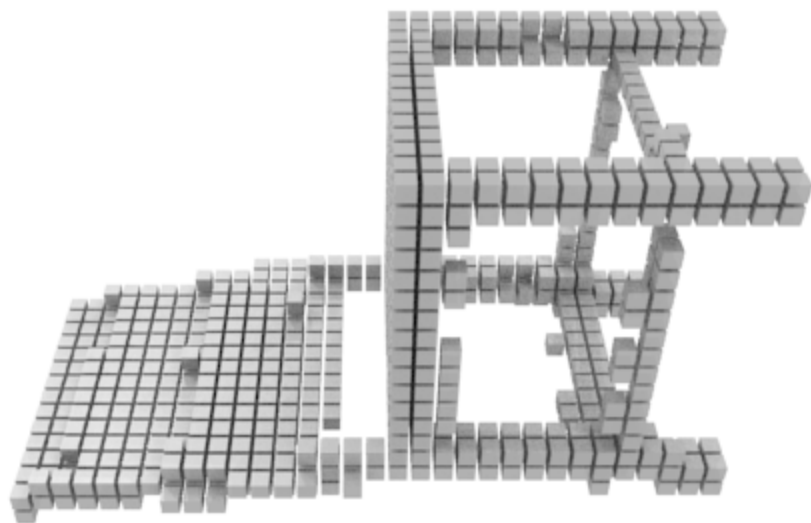

Supplement: Supplemental Information 4 [file peerj-cs-10-2403-s004.zip › ResultFromOurMethod/cs-100114-R3DSWINchair.pdf]

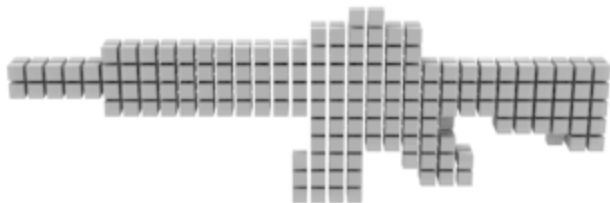

Supplement: Supplemental Information 4 [file peerj-cs-10-2403-s004.zip › ResultFromOurMethod/cs-100114-R3dswinrifle.pdf]

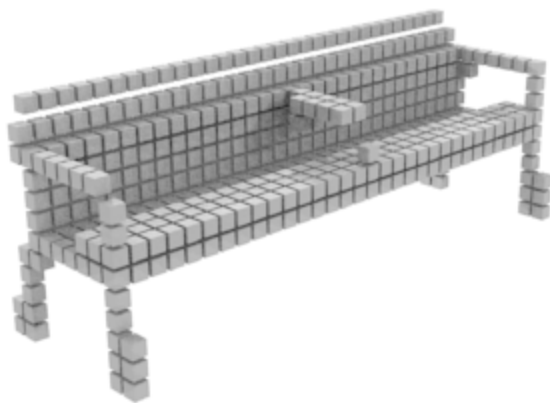

Supplement: Supplemental Information 4 [file peerj-cs-10-2403-s004.zip › ResultFromOurMethod/cs-100114-R3dswin_longchair.pdf]

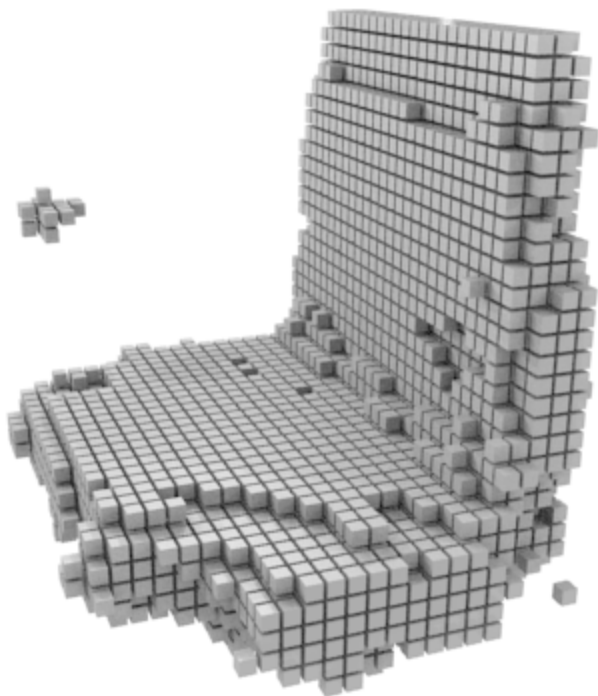

Supplement: Supplemental Information 5 [file peerj-cs-10-2403-s005.zip › result_from_umiformer/cs-100114-pix3d_UMIFormer0.pdf]

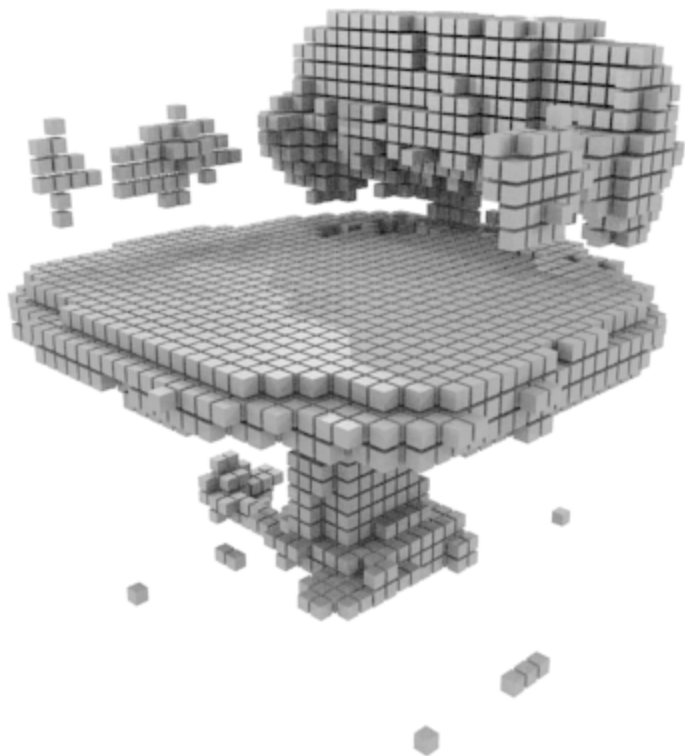

Supplement: Supplemental Information 5 [file peerj-cs-10-2403-s005.zip › result_from_umiformer/cs-100114-pix3d_UMIFormer1.pdf]

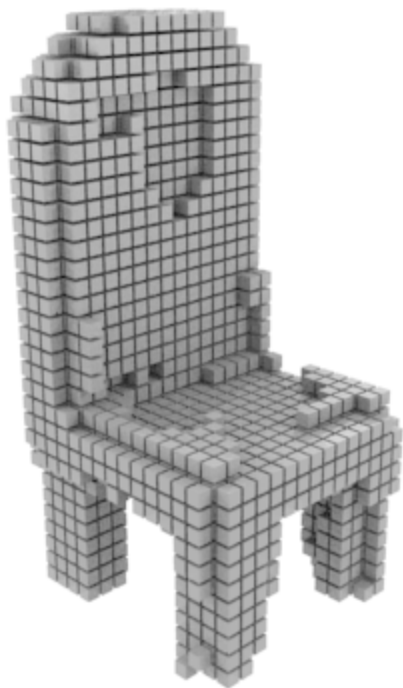

Supplement: Supplemental Information 5 [file peerj-cs-10-2403-s005.zip › result_from_umiformer/cs-100114-UMIFormer3b3a9f4e3aa9f2f4d39a194653571dfc.pdf]

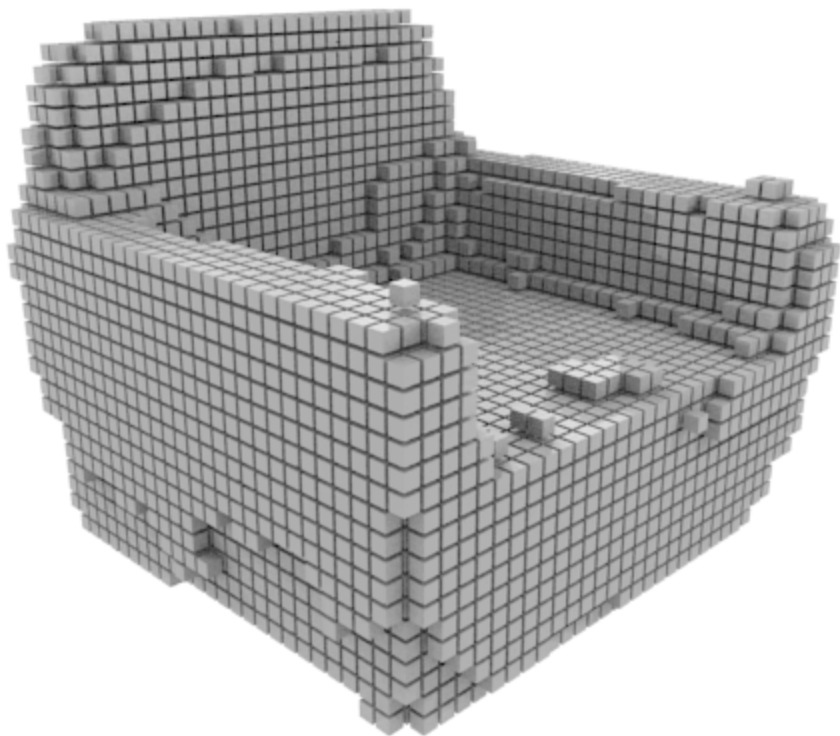

Supplement: Supplemental Information 5 [file peerj-cs-10-2403-s005.zip › result_from_umiformer/cs-100114-UMIFormerbe4c88a130e622a21961e650f3cfa396.binvox.pdf]

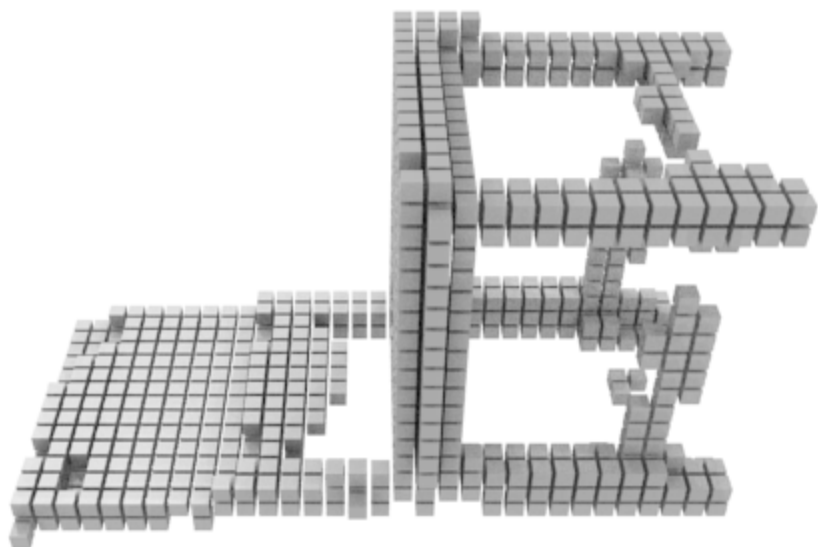

Supplement: Supplemental Information 5 [file peerj-cs-10-2403-s005.zip › result_from_umiformer/cs-100114-UMIFormerchair.pdf]

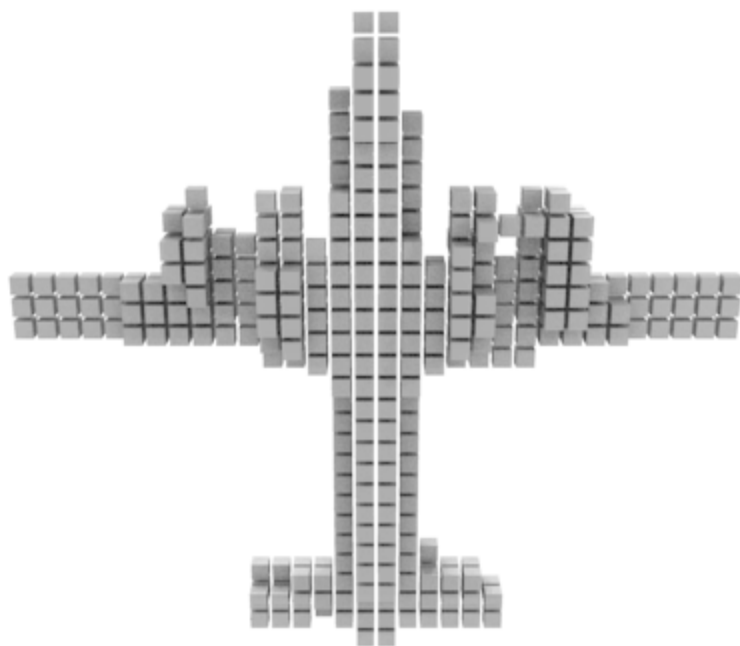

Supplement: Supplemental Information 5 [file peerj-cs-10-2403-s005.zip › result_from_umiformer/cs-100114-UMIformerplane.pdf]

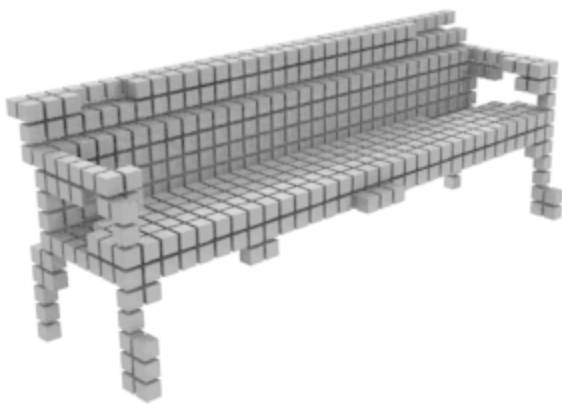

Supplement: Supplemental Information 5 [file peerj-cs-10-2403-s005.zip › result_from_umiformer/cs-100114-UMIFORMER_longchair.pdf]

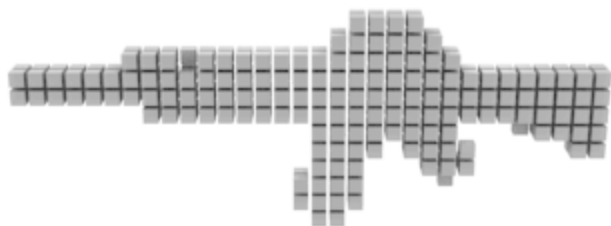

Supplement: Supplemental Information 5 [file peerj-cs-10-2403-s005.zip › result_from_umiformer/cs-100114-UMIFormer_rilfe.pdf]
